# Immune profiling of pediatric germ cell tumors identifies key cell populations and novel therapeutic targets

**DOI:** 10.3389/fimmu.2025.1579948

**Published:** 2025-06-20

**Authors:** Lenilson Silva, Ingridy Izabella Vieira Cardoso, Marcelo Cavalcanti da Cruz, Thaíssa Maria Veiga Faria, Gisele Eiras Martins, Bruna Minniti Mançano, Luiz Fernando Lopes, Rui Manuel Reis, Daniel Antunes Moreno, Mariana Tomazini Pinto

**Affiliations:** ^1^ Molecular Oncology Research Center, Barretos Cancer Hospital, São Paulo, Brazil; ^2^ Pediatric Oncology Research Group (GPOPed), Molecular Oncology Research Center, Barretos Cancer Hospital, São Paulo, Brazil; ^3^ Department of Pathology, Barretos Cancer Hospital, São Paulo, Brazil; ^4^ Children’s Cancer Hospital, Barretos Cancer Hospital, São Paulo, Brazil; ^5^ Life and Health Sciences Research Institute (ICVS), Medical School. University of Minho, Braga, Portugal

**Keywords:** germ cell tumors, pediatric tumor, immune profiling, immune checkpoint, tumor immunology

## Abstract

**Introduction:**

Pediatric germ cell tumors (GCTs) are rare malignancies, comprising only about 3% of childhood cancers. Despite surgery and platinum-based chemotherapy being mainstays of treatment, their effectiveness varies by tumor subtype, and long-term toxicities remain a concern. We therefore explored the immune landscape of pediatric GCTs to uncover subtype-specific immunological features and identify potential immunotherapeutic targets.

**Methods:**

This retrospective study investigated the immune landscape of pediatric GCTs, utilizing a cohort of 17 patients, including 14 extracranial GCTs (11 ovarian, 3 testicular), three central nervous system (CNS) mixed tumors and four non-neoplastic tissues (controls).

**Results:**

Immune profiling revealed distinct immune microenvironments across the GCT subtypes. Dysgerminomas exhibited an immune-active profile with elevated levels of T cells, CD8^+^ T cells, and cytotoxic cells, alongside upregulation of immune checkpoints *CTLA4*, *TIGIT*, and *IDO1*, suggesting potential responsiveness to checkpoint inhibitors. In contrast, yolk sac tumors displayed an immunosuppressive environment with high *CD24* and *PVR* expression, indicative of unique immune evasion mechanisms. Embryonal carcinomas also showed high *CD24* expression. An *in silico* analysis of adult GCTs highlighted similarities and differences with pediatric cases; *IDO1* and *CD24* were consistently upregulated across age groups, while *CTLA4* and *PVR* showed variation.

**Conclusion:**

Overall, this study provides new insights into pediatric GCT immunology, supporting the potential for tailored immunotherapeutic strategies targeting the distinct immune profiles of pediatric GCT histologies.

## 1 Introduction

Pediatric germ cell tumors (GCTs) are a heterogeneous group of rare malignancies arising from primordial germ cells, predominantly affecting the gonads but also occurring in extragonadal locations such as the central nervous system (CNS) (1). GCTs are classified into distinct histological subtypes, each with unique biological characteristics and clinical behaviors. Malignant subtypes include seminoma (testicular); dysgerminoma (ovarian); yolk sac tumor (YST); embryonal carcinoma (EC); choriocarcinoma, and mixed tumors, while teratomas can be either benign or malignant depending on their differentiation and location (2, 3). Pediatric GCTs represent only about 3% of all childhood cancers (4). Although rare, these tumors pose significant clinical challenges due to their histological diversity and variable responses to treatment.

The primary treatment for pediatric GCTs typically involves surgery and platinum-based chemotherapy, which can cause significant short—and long-term toxicities and affect patients’ quality of life (5). Moreover, treatment options for relapsed or refractory GCTs remain limited, underscoring the need for novel therapeutic approaches that not only improve outcomes but also reduce treatment-related adverse effects (6, 7).

Recent advances in cancer immunotherapy have highlighted the potential of the immune microenvironment to influence tumor behavior and therapeutic response (8, 9). Immune checkpoint inhibitors, which have shown significant success in various adult cancers, work by targeting immune checkpoints (10–12). While immune checkpoint therapies showed promising results for many cancer types, their efficacy in pediatric GCTs remains largely unexplored. A comprehensive understanding of the immune landscape in pediatric GCTs, including immune cell composition and checkpoint expression, is critical for identifying potential therapeutic targets and designing tailored immunotherapy approaches.

This study evaluated the gene expression patterns and immune cell landscapes across pediatric GCT subtypes, identifying subtype-specific immune microenvironments and key immune checkpoints involved in immune evasion. We validated our findings using an adult male GCT dataset to explore age-related immune regulation. The analysis aimed to uncover unique immune signatures and mechanisms of immune escape, providing insights for developing targeted immunotherapies for pediatric GCTs.

## 2 Methods

### 2.1 Study population

This retrospective study included chemotherapy-naïve tumor samples from 17 pediatric GCTs patients treated at Barretos Children’s Cancer Hospital (Brazil) between 2000 and 2021. It included 14 extracranial tumors (11 ovarian, 3 testicular) and three CNS mixed tumors. GCT classification adhered to the 2016 World Health Organization (WHO) guidelines. Four adjacent non-tumoral tissues, including two ovarian and two testicular, were used as controls. Clinicopathological data were retrospectively collected from patient medical records. This study received approval from the local ethics committee, CEP-HCB (Barretos Cancer Hospital IRB/Project No. 2258/2021). Given the substantial challenge in reaching participants for consent—particularly as a portion of this population is deceased—the CEP-HCB granted an exemption from requiring informed consent. This decision considered the potential for significant emotional distress to surviving family members. Additionally, as a retrospective study, the research was limited to the examination of pre-existing slides and paraffin blocks stored in the Barretos Cancer Hospital’s pathology department, along with a review of medical records. Therefore, since the requirement for informed consent was waived by the CEP-HCB, patients did not provide written or verbal informed consent.

### 2.2 RNA isolation

Total RNA was extracted from formalin-fixed paraffin-embedded (FFPE) tumor samples that were sectioned and mounted onto slides at a thickness of 10μm. Tumor area was demarcated on slides by an experienced pathologist, based on H&E staining, to ensure at least 70% tumor content. A control group was established by using non-tumoral tissue adjacent to the tumors from four pediatric GCT patients. RNA was isolated using the RecoverAll Total Nucleic Acid Isolation Kit (Thermo Fisher Scientific), following the manufacturer’s instructions, and as reported (13). The purity and concentration of total RNA were assessed using NanoDroPVR 2000 (Thermo Fisher Scientific) and Qubit 2.0 instrument (Thermo Fisher Scientific).

### 2.3 Immune gene expression analysis

The nCounter PanCancer Immune Profiling Panel was employed for gene expression analysis, targeting more than 700 genes involved in immune response (NanoString Technologies), using the nCounter Analysis System as previously reported (14, 15). Briefly, a total of 300 ng of RNA per sample was used for the assay. Hybridization was performed for 24 hours at 65°C, after which the samples were loaded onto the nCounter Prep Station for binding and washing. The samples were then scanned, capturing 550 fields of view using the nCounter Digital Analyzer (NanoString Technologies).

Data was analyzed using ROSALIND^®^. Quality control checks were performed on imaging quality, binding density, detection limits, positive controls, and housekeeping gene counts to ensure data integrity. Only samples with more than 25% of housekeeping genes above 50 counts were included in the analysis. Normalization with housekeeping genes was performed based on the geNorm algorithm (16). The abundance of various cell populations was calculated on ROSALIND using the Nanostring Cell Type Profiling Module. A fold change threshold of ±1.5 was used for differentially expressed genes (DEGs), and the p-value adjustment was performed using the Benjamini-Hochberg method of estimating false discovery rates (FDR) with a significance level set at p ≤ 0.05. The STRING algorithm was used to predict protein-protein association networks and perform functional enrichment analyses (17).

### 2.4 *In silico* analysis

For the *in silico* analysis, the R2: Genomics Analysis and Visualization Platform (http://r2.amc.nl) was employed using the Korkola et al. gene expression dataset (GSE3218, Affymetrix U133 microarray platform) (18). The dataset comprises 101 adults male GCTs, including both primary and metastatic samples: 42 mixed tumors, 17 seminomas, 15 EC, 15 teratomas, 10 YST, two choriocarcinomas, and six normal testis specimens from similarly aged patients, including one pooled normal testis sample. The mixed GCT specimens were classified according to their predominant histology. One mixed tumor sample was excluded from the analysis due to the lack of histological information.

### 2.5 Statistical analysis

Heatmaps with hierarchical clustering were generated using the ComplexHeatmap R package v2.20.0 (19). The clustering was performed using Pearson’s correlation distance to measure sample similarity. The Kruskal-Wallis test, corrected with the Benjamini-Hochberg method, was used for multiple comparisons, with FDR-adjusted p-values ≤ 0.05 considered as a discovery. Dunn’s *post hoc* test was performed using the dunn.test R package v1.3.6, and boxplots were generated using the ggpubr R package v0.6.0.

## 3 Results

### 3.1 Clinicopathologic features of pediatric GCTs patients

The clinicopathologic features of the 17 patients are summarized in **Table 1**. The average age at diagnosis was 12.4 years (range, 0.7-18 years), with an average of 14 years for females and 8.5 years for males. Most patients had ovarian tumors (n=11, 64.70%), three patients (17.65%) had testicular tumors, and three patients (17.65%) had CNS tumors. Clinical findings showed that 35.3% (6/17) of patients had dysgerminomas, followed by 29.4% (5/17) yolk sac tumors, 17.65% (3/17) embryonal carcinomas, and 17.65% (3/17) CNS mixed tumors. Most of the patients were treated with chemotherapy (76.5% - 13/17) and were still alive (76.5% - 13/17).

**Table 1:** Clinical characteristics of pediatric patients.

Features	Total, *n* (%)
N (%)	17 (100%)
Age (average)	12.4 (0.7-18 years)
Sex	
Male	5
Female	12
Tumor location	
Ovary	11 (64.7%)
Testis	3 (17.65%)
CNS	3 (17.65%)
Histology	
Dysgerminoma	6 (35.3%)
Yolk sac tumor	5 (29.4%)
Embryonal carcinoma	3 (17.65%)
CNS mixed tumor	3 (17.65%)
Germinoma, teratoma	1
Embryonal carcinoma, yolk sac tumor, teratoma	1
Teratoma, embryonal carcinoma, yolk sac tumor, germinoma	1
Chemotherapy	
Yes	13 (76.5%)
No	4 (23.5%)
Status	
Alive	13 (76.5%)
Dead	4 (23.5%)

CNS, Central Nervous System.

### 3.2 Differential immune expression across pediatric GCTs

**Figure 1:**
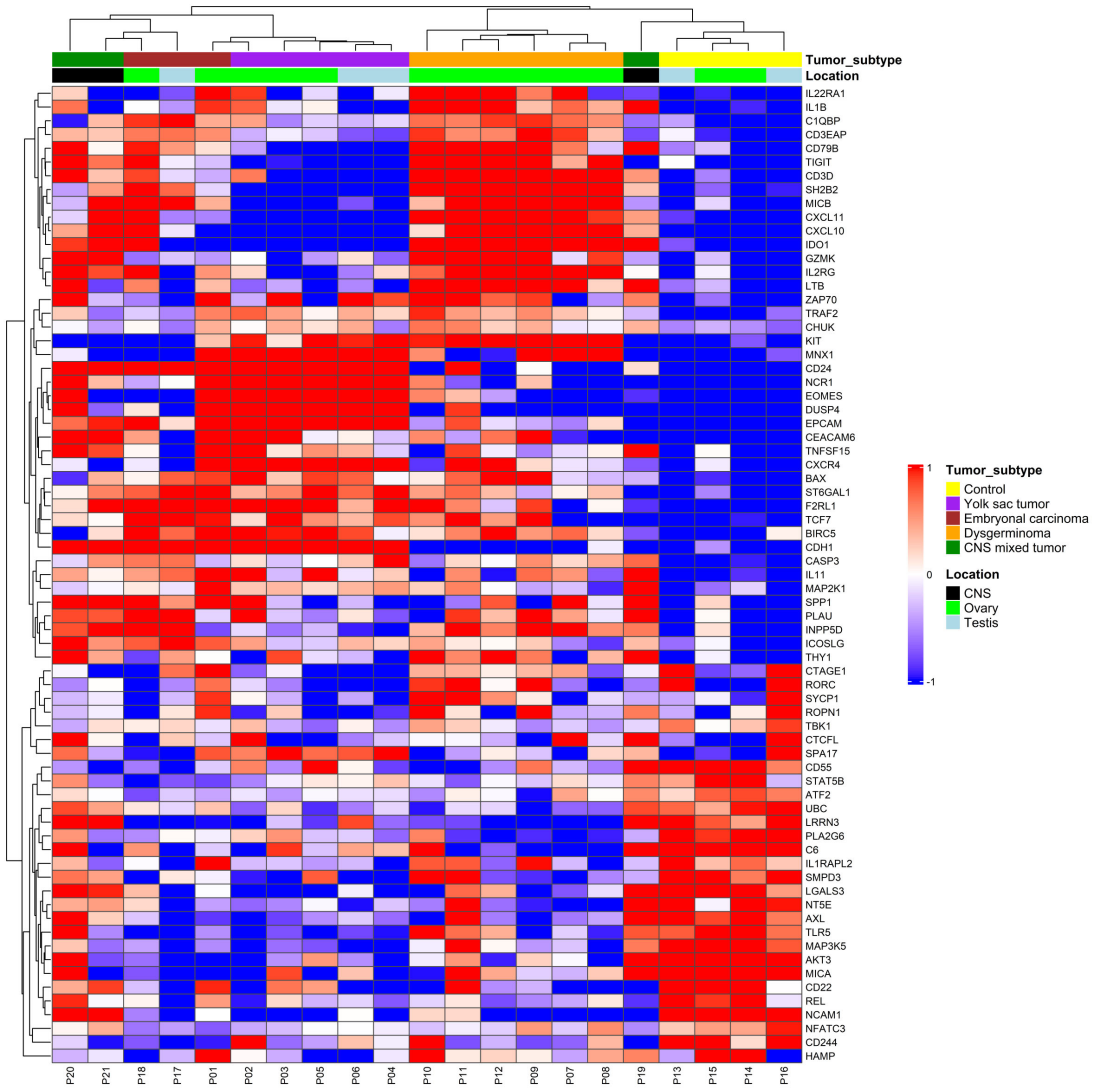
Immune gene expression profile in pediatric germ cell tumors. Expression levels were used to group gene profiles according to their similarities between histological types, including yolk sac tumor (purple), embryonal carcinoma (red), dysgerminoma (orange), and central nervous system (CNS) (green). The two-color heatmap displays the mean-subtracted normalized log2 expression values for each gene across different samples. Blue and red colors indicate downregulation and upregulation, respectively, relative to the control group. Unsupervised hierarchical clustering was performed based on immune gene expression, revealing distinct immune-related gene patterns across different tumor subtypes.

We were able to perform the immune expression analysis in all cases. We identified 71 genes differentially expressed when compared to the control group, with most tumor samples forming clear clusters distinct from the controls, except a CNS mixed tumor sample (**Figure 1**). Unsupervised hierarchical clustering of immune gene expression profiles across pediatric GCT revealed two main tumor clusters, each with distinct immune gene expression patterns. Cluster A includes CNS mixed tumor, EC, YST, while cluster B was primarily composed of dysgerminomas. Notably, two CNS mixed tumors clustered closely with ECs due to a predominant EC component, one EC sample grouped with YSTs, and another CNS mixed tumor, containing germinoma and teratoma components, clustered with the control group. Additionally, the identified differentially expressed genes can characterize distinct profiles for various histological types of GCTs, regardless of the primary site (ovary, testis, or CNS).

### 3.3 Pediatric GCTs exhibit distinct histology-specific immune signatures

**Figure 2:**
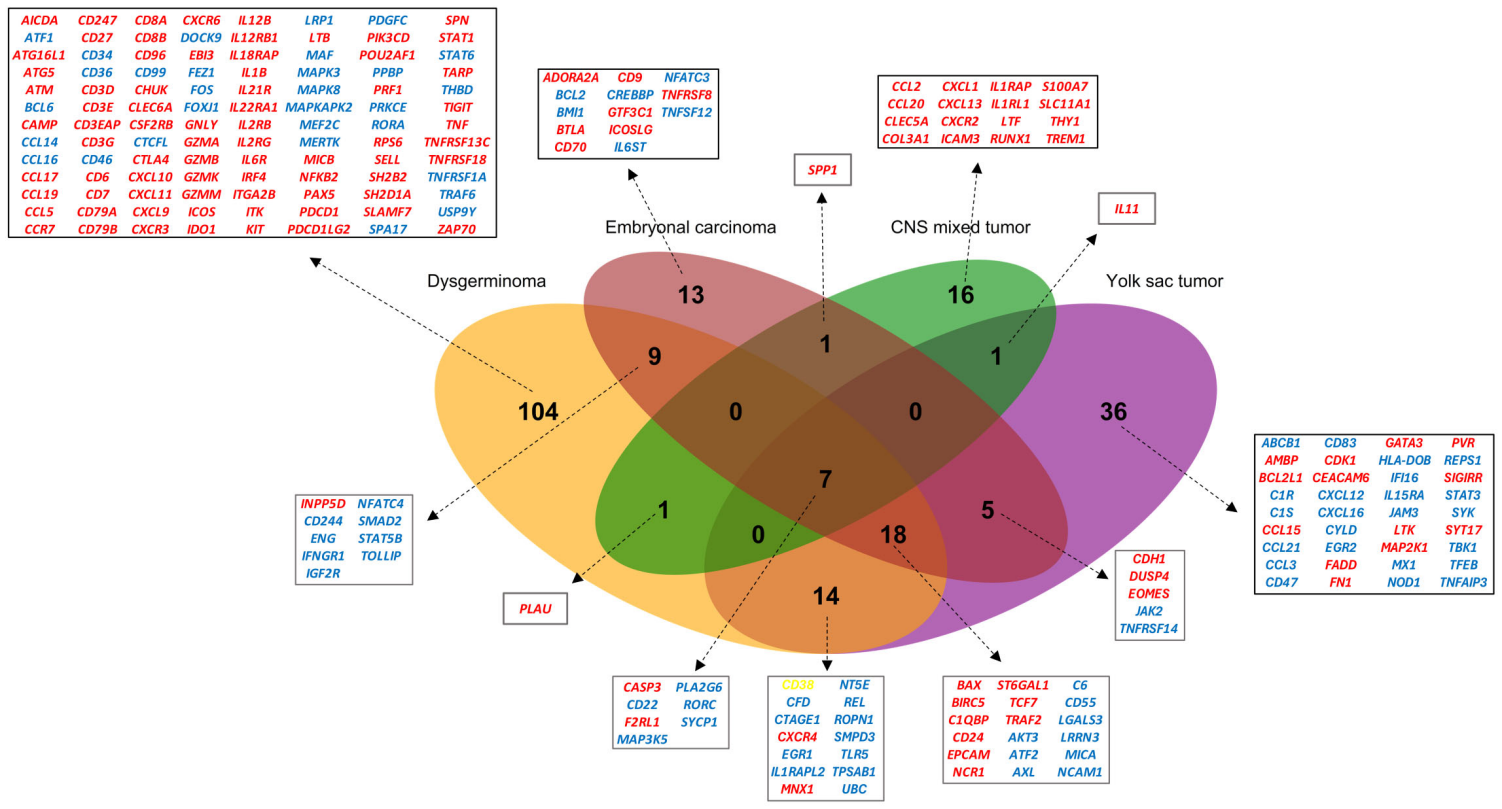
Overlap of differentially expressed genes (DEGs) in pediatric germ cell tumors. Venn diagram illustrating the overlap of DEGs across four pediatric GCT subtypes (dysgerminoma, embryonal carcinoma, CNS mixed tumor, and yolk sac tumor). Genes are color-coded to indicate expression patterns: red represents upregulated genes, blue indicates downregulated genes and yellow highlights genes upregulated in dysgerminoma but downregulated in yolk sac tumors. CNS, Central nervous system.

Individual comparisons between each histology and the control group were conducted, illustrating both overlapping and unique gene expression patterns for each histological subtype (**Figure 2**). Dysgerminomas showed the highest number of differentially expressed genes (153 DEGs), with 90 upregulated and 63 downregulated genes. YST had 81 DEGs (30 upregulated, 51 downregulated), EC had 53 DEGs (23 upregulated, 30 downregulated), and CNS mixed tumor had 26 DEGs (21 upregulated, 5 downregulated). Dysgerminomas also exhibited the highest number of unique immune signatures (104 genes), followed by YST (36 genes), EC (13 genes), and CNS mixed tumor (16 genes).

Several immune-related genes were shared across tumor types, with seven genes (*CASP3*, *F2RL1*, *CD22*, *RORC*, *PLA2G6*, *SYCP1*, *MAP3K5)* common to all four histologies (**Figure 2**). Among these, 25 genes, including the seven shared across all histologies, were common among gonadal tumors, with 18 being unique to them.

**Figure 3:**
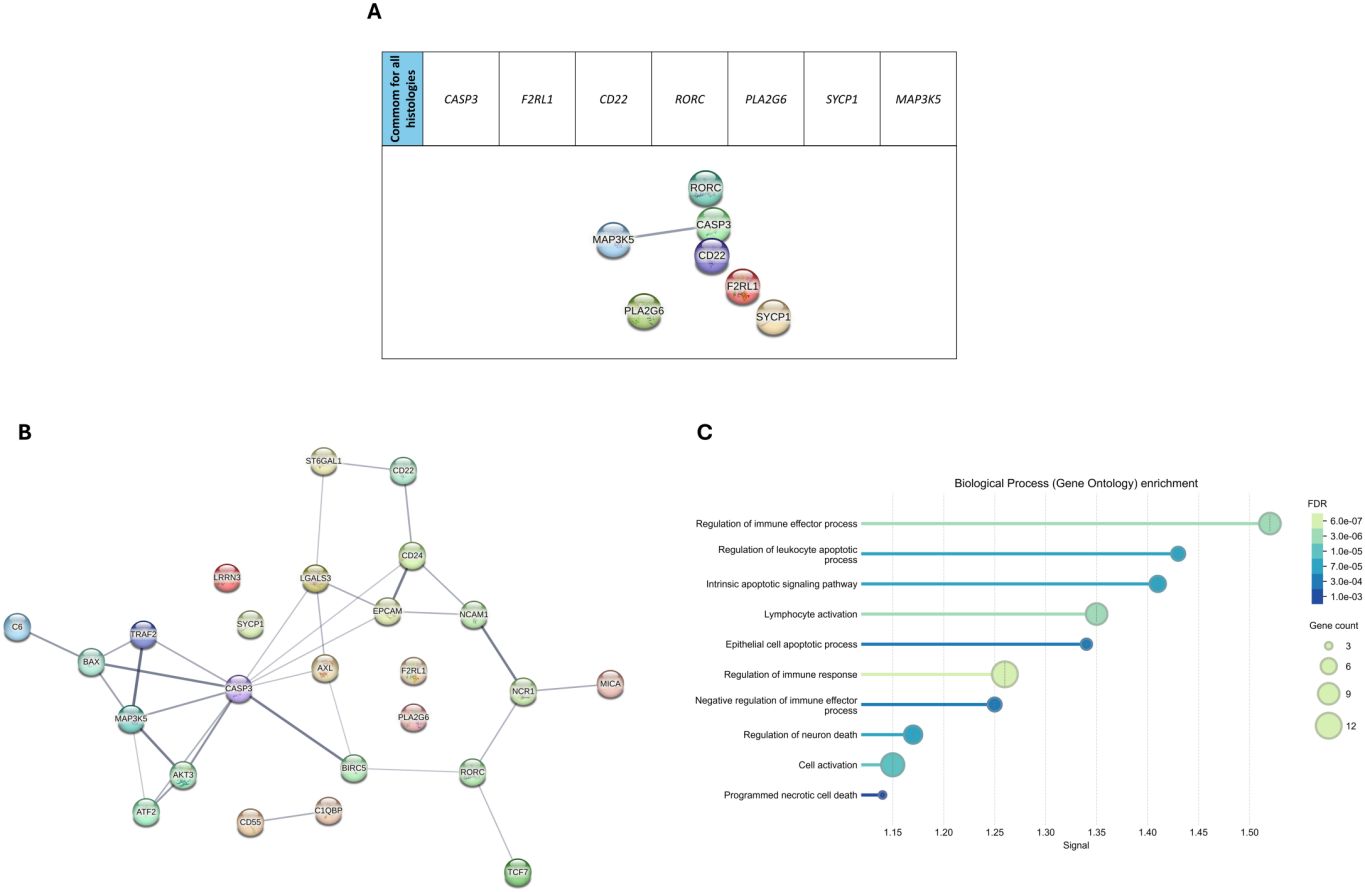
Pathway analysis in pediatric germ cell tumors. **(A)** STRING protein-protein interaction (PPI) network of the seven genes shared across all four tumor subtypes. **(B)** Expanded PPI network for differentially expressed genes (DEGs) in gonadal tumors, illustrating key interactions among target proteins. **(C)** Biological process enrichment analysis (Gene Ontology) of the DEGs. The size of the dots represents gene count per pathway, and the color gradient indicates the significance level of the false discovery rate (FDR) for each enriched process.

We used the STRING algorithm to predict protein-protein association networks and perform functional enrichment analyses to explore the functional relevance of the DEGs in gonadal tumors. The initial network of the seven common genes for all histologies did not show strong interaction, suggesting limited direct connectivity among these core immune genes (**Figure 3A**). In contrast, the network of the 25 genes shared among gonadal tumors revealed significant interactions, indicating potential coordinated immune pathways specific to gonadal GCTs (**Figure 3B**). Functional enrichment analysis highlighted processes such as immune effector regulation, apoptotic pathways, and cell activation, suggesting that these genes are involved in pathways critical to pediatric GCT tumorigenesis and immune response (**Figure 3C**).

### 3.4 Immune cell composition highlights unique microenvironments in pediatric GCTs

**Figure 4:**
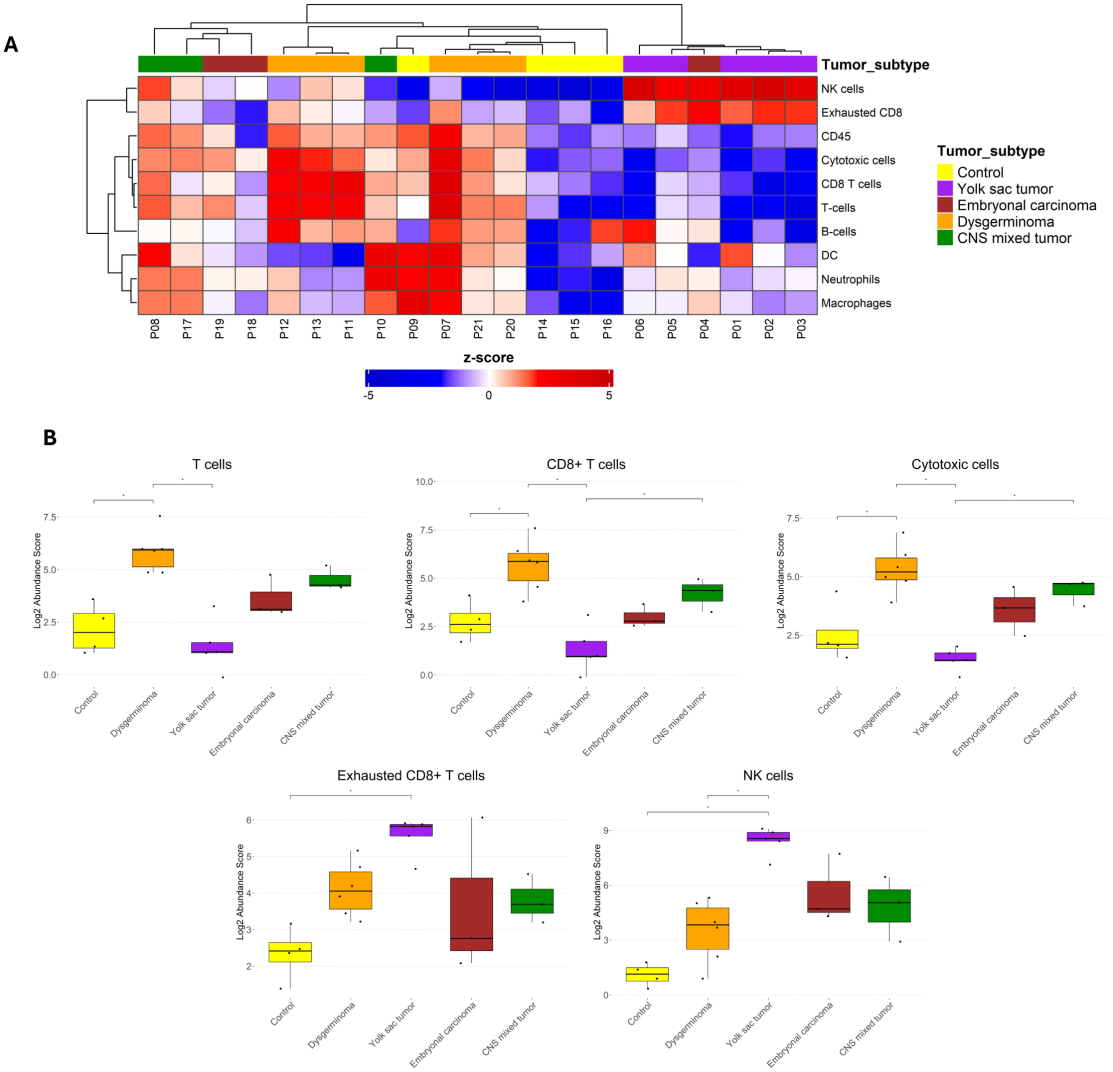
Immune cell abundance in pediatric germ cell tumor subtypes. **(A)** Heatmap displaying the z-scores of immune cell abundance for ten distinct immune cell types across different pediatric GCT subtypes and control samples. Blue and red colors indicate downregulation and upregulation, respectively, relative to the control group. **(B)** Boxplots comparing immune cell abundance scores with significant differences among the groups for selected immune cell populations. Statistical significance is indicated by asterisks (*p ≤ 0.05, FDR-adjusted).

To better understand the immune microenvironment in pediatric GCTs, we analyzed the composition of immune cells across tumor subtypes using the cell type score based on specific immune cell-type mRNA expression. Gene expression signatures for individual cell types identified ten distinct immune cell types: T cells, CD8^+^ T cells, cytotoxic cells, exhausted CD8^+^ T cells, natural killer (NK) cells, B cells, neutrophils, macrophages, dendritic cells (DC), and CD45^+^ cells. Unsupervised hierarchical clustering revealed a relative separation among tumor subtypes, indicating distinct immune cell compositions across different histologies (**Figure 4A**).

When comparing immune cell abundances among the groups, dysgerminoma exhibited a significantly higher abundance of T cells, CD8^+^ T cells, and cytotoxic cells than the control group (**Figure 4B**). This elevated presence of T cells, particularly CD8^+^ cytotoxic cells, suggests an active immune response within dysgerminoma, potentially indicating a more immunologically reactive environment capable of targeting tumor cells. In contrast, YST showed an increased abundance of exhausted CD8^+^ T cells and NK cells. The presence of exhausted CD8^+^ T cells suggest a state of chronic antigen exposure or immune suppression within YST, possibly indicating an immune environment where the anti-tumor activity of T cells is impaired.

### 3.5 Immune checkpoint expression across pediatric and adult GCTS

We analyzed the upregulation of immune checkpoint genes among the DEGs for each histological type (**Supplementary Figure 1**). *TIGIT*, *IDO1*, *CTLA4*, and *PDCD1* were specifically upregulated in dysgerminomas. *CD24* was shared between YST and EC, but *PVR* was uniquely upregulated in YST, while *BTLA* was upregulated explicitly in EC.

**Figure 5:**
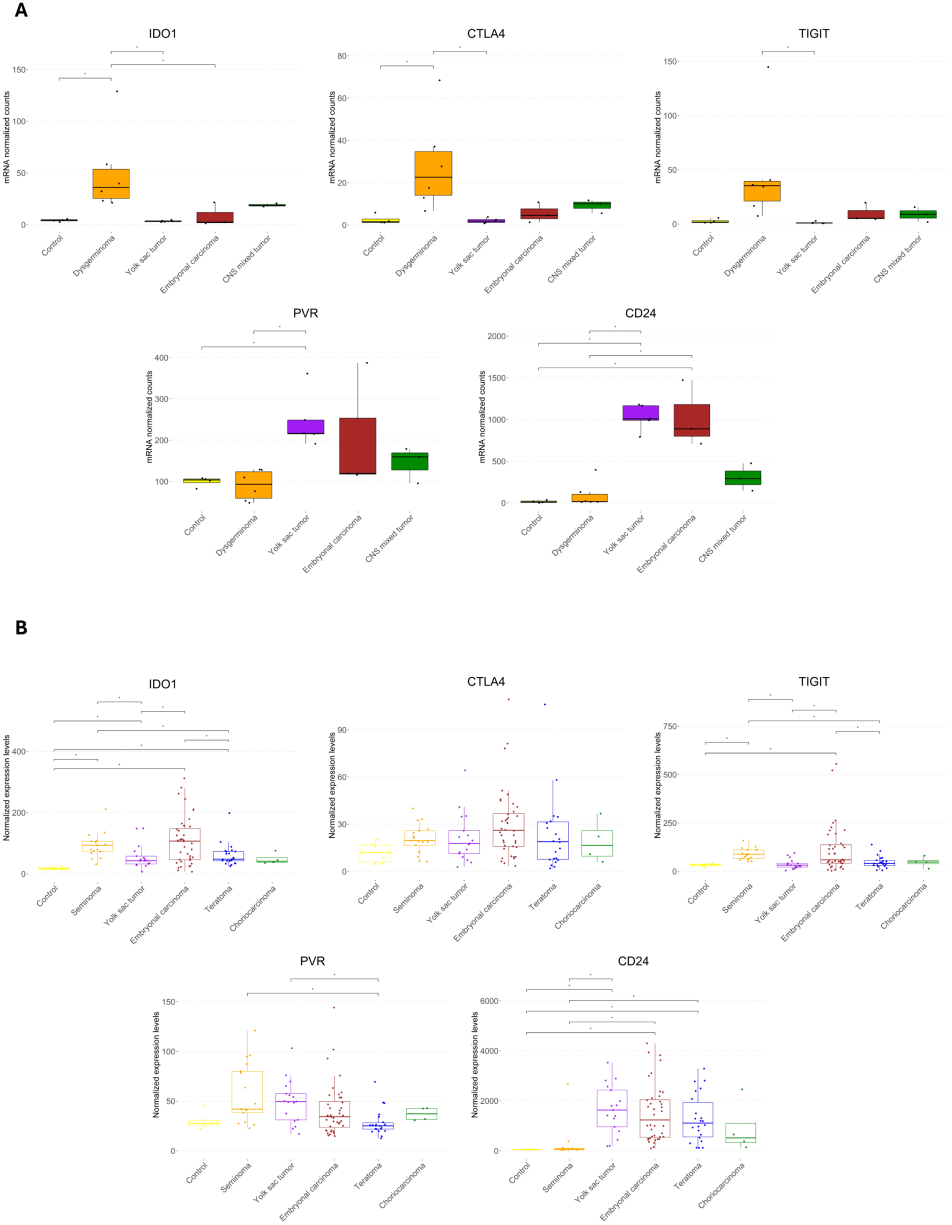
Expression of immune checkpoint genes across pediatric and adult germ cell tumor subtypes. **(A)** Boxplots showing the normalized mRNA counts for immune checkpoint genes (*IDO1*, *CTLA4*, *TIGIT*, *PVR*, and *CD24*) across pediatric GCT histologies and control samples. **(B)** Boxplots displaying normalized expression levels of the same immune checkpoint genes in an *in silico* analysis of adult male germ cell tumors. Statistical significance is indicated by asterisks (*p ≤ 0.05, FDR-adjusted).

When we compared the normalized counts of these immune checkpoints across the pediatric GCT subtypes (**Figure 5A**), we found that dysgerminoma showed significantly higher expression of *IDO1* and *CTLA4* than the control group. *TIGIT* was also upregulated in dysgerminomas, although it did not show significant normalized counts compared to the control. YST exhibited high levels of *CD24* and *PVR*, while ECs showed higher levels of *CD24* compared to the control group.

To determine whether similar immune checkpoint expression patterns are observed in adult GCTs, we conducted an *in silico* analysis on a dataset comprising 100 male GCT samples and six normal samples (**Figure 5B**). This comparison revealed that *IDO1* was highly expressed in seminoma, YST, EC, and teratoma compared to controls, suggesting a common role for *IDO1* in creating an immunosuppressive environment across both pediatric and adult GCTs. However, *CTLA4* did not show any significant difference in adult GCTs. *TIGIT* showed elevated levels in adult seminoma and EC, similar to its upregulation in pediatric dysgerminomas, suggesting it could be a relevant checkpoint in immune suppression in both age groups for these histologies. *PVR* did not differ significantly from the control group in adult GCTs, contrasting with its elevated expression in pediatric YSTs. *CD24* was highly expressed in adult YST, EC, and teratoma, aligning with its elevated levels in pediatric YST and EC, indicating a conserved immune escape mechanism through *CD24* across age groups.

## 4 Discussion

This was the first study to profile the immune landscape of pediatric germ cell tumors across various histological subtypes, shedding light on both shared and unique immune features within these tumors. We described the immune cell type abundance across pediatric GCTs, with unsupervised hierarchical clustering, highlighting distinct compositions of the tumor microenvironment within each GCT subtype. Dysgerminomas exhibited significantly higher abundances of T cells, CD8^+^ T cells, and cytotoxic cells, indicating a relatively immune-active microenvironment that may support anti-tumor responses. Of note, this subtype also displayed upregulation of immune checkpoints such as *CTLA4*, *TIGIT*, and *IDO1*. Although we did not observe differential expression of *PD-L1*, prior studies reported high *PD-L1* expression in dysgerminomas, correlating with dense tumor-infiltrating lymphocytes (TIL), including cytotoxic CD8^+^ T cells (20). Similar results were found in adult seminomas, where high levels of *PD-1* and *TIGIT* were detected on infiltrating T cells, where increased cytotoxic T cell infiltration is associated with favorable outcomes and potential responsiveness to immunotherapy (21, 22). The expression of such checkpoints highlights a balance of immune activation and suppression, suggesting that pediatric dysgerminomas could be responsive to immunotherapies targeting multiple immune checkpoints to unlock anti-tumor immune potential.

Yolk sac tumors, on the other way, displayed an increased abundance of exhausted CD8^+^ T cells and NK cells, suggesting an immunosuppressive tumor microenvironment (TME). The exhausted T cells indicate chronic antigen exposure, likely impairing effective anti-tumor activity. While indicative of innate immune activation, NK cells may be dysregulated or insufficient in countering immune evasion in YSTs, as seen in other tumor types where NK cell function is hindered by the immunosuppressive TME (23–25). In support of this, studies across various cancers have demonstrated that NK cells rapidly lose their cytotoxic functions after entering the tumor, adopting phenotypic states associated with tissue residency and functional impairment (26, 27). In gastric cancer liver metastases, NK cells showed reduced expression of *IFNγ* and *TNF*, partially driven by TGFβ-mediated suppression (28). A similar mechanism was observed in metastatic breast cancer, where *TGFβ* altered NK cell metabolism and led to mitochondrial dysfunction, which was reversible upon *TGFβ* blockade (29). Mitochondrial fragmentation has also been linked to NK cell dysfunction in liver cancer, with excessive Drp1-mediated mitochondrial fission impairing their cytotoxic potential (30). Moreover, oxidative stress and nutrient deprivation in the TME can suppress NK cell metabolism, leading to reduced glycolysis, oxidative phosphorylation, and effector function (31). Although our study did not assess NK cell activity directly, these converging findings from solid tumors support the hypothesis that NK cells in YSTs may similarly exist in a phenotypically altered, less cytotoxic state.

YST and EC exhibited elevated levels of *CD24*, a molecule linked to immune evasion and chemoresistance (32–34). The findings align with a recent study, which showed that *CD24* supports an undifferentiated, pluripotent cell state in ECs and that blocking *CD24* with a monoclonal antibody can enhance cisplatin sensitivity *in vitro*, even in cisplatin-resistant clones (35). This suggests that CD24-targeted therapies might potentiate cisplatin efficacy in pediatric GCTs, especially in resistant cases.

Additionally, *PVR* was upregulated in pediatric YST. *PVR*, a member of the nectin-like family, is increasingly recognized for its role in promoting tumorigenic processes such as proliferation, migration, and immune suppression (36). High *PVR* expression correlates with poor prognosis and increased invasiveness, as observed in hepatocellular carcinoma and lung squamous cell carcinoma, where elevated *PVR* levels are associated with decreased overall and recurrence-free survival rates (37, 38). In pancreatic cancer, intratumoral NK cells show downregulation of activating receptors such as DNAM-1 and NKp30 and increased expression of inhibitory ligands like PVR on tumor cells, impairing their function despite infiltration​ (39).

In the present study, the CNS mixed tumors presented the lowest number of DEGs and no significant immune cell abundance or checkpoint expression at the mRNA level. This result may stem from the small sample size or the heterogeneous composition of mixed tumors. However, previous studies on CNS GCTs reported high *PD-L1* expression associated with CD8^+^ T cell infiltration in tumors such as germinomas, suggesting a partially active immune environment despite limited response to immune checkpoint inhibitors (40, 41).

Our *in silico* comparison of immune checkpoint expression in pediatric and adult GCTs revealed that *IDO1* and *CD24* were elevated across age groups, yet *CTLA4* and *PVR* checkpoints showed variations. *PVR* was particularly upregulated in pediatric YST, suggesting differences in immune evasion mechanisms between pediatric and adult GCTs. *CTLA4* did not show significant differences overall in adult GCTs; however, *CTLA4* immunostaining was reported in adult testicular YST, choriocarcinoma, and teratoma, where it was linked to an immunosuppressive environment (42). This finding suggests that *CTLA4* may have a subtype-specific role in GCTs, with its upregulation in pediatric dysgerminomas potentially indicative of a similar immunosuppressive function.

Despite our findings, this study exhibited some limitations. The sample size for pediatric GCTs was limited, which may affect the generalizability of the findings across all pediatric GCT subtypes. Some GCT histologies, including choriocarcinoma and seminoma, were not represented. Immune system maturation and immune cell dynamics can differ significantly across this developmental spectrum (43). However, due to the small sample size, we were unable to stratify or statistically evaluate age-related immune variation. Additionally, our analysis focused on mRNA expression levels, which may not directly correlate with protein expression or functional activity of immune checkpoints in the tumor microenvironment. Further studies using proteomic approaches and single-cell analyses would be beneficial in providing a more comprehensive view of the immune landscape in pediatric GCTs.

Overall, our study reveals promising new therapeutic targets for pediatric GCTs. In dysgerminomas, targeting *IDO1* and *CTLA4* could enhance T cell-mediated anti-tumor responses. For YST and EC, *CD24* inhibition combined with chemotherapy may overcome chemoresistance, while *PVR*-targeted therapies could reduce immune evasion in YST. Age-specific immunotherapy approaches are critical, as checkpoint expression differs between pediatric and adult GCTs. While some checkpoints, like *IDO1* and *CD24*, are relevant across age groups, others, such as *CTLA4*, may play subtype-specific roles that vary with age. Further research is needed to optimize checkpoint inhibitor combinations and immune-modulatory therapies for pediatric GCTs.

In conclusion, this work highlights specific immune checkpoint genes upregulated across different histological types of GCTs, offering insights into potential therapeutic targets. The unique expression profiles identified may contribute to a more personalized approach to GCT treatment, although further validation through prospective, multicenter cohort studies is essential to confirm these findings. While numerous clinical trials are already exploring combinations of immune checkpoint inhibitors with standard therapies in adult cancers, pediatric trials still need to be expanded. Establishing the safety and efficacy of these combinations in children can transform treatment strategies for childhood cancers that are currently nonresponsive to existing therapies, ultimately leading to improved patient outcomes.

## Data Availability

The original contributions presented in the study are publicly available. This data can be found at Gene Expression Omnibus (https://www.ncbi.nlm.nih.gov/geo/query/acc.cgi?acc=GSE297926; accession number GSE297926).

## References

[1] Pierce JL Frazier AL Amatruda JF. Pediatric germ cell tumors: A developmental perspective. Adv Urol. (2018) 2018:1–8. doi: 10.1155/2018/9059382 PMC5817207 29515628

[2] Moch H Amin MB Berney DM Compérat EM Gill AJ Hartmann A. The 2022 world health organization classification of tumours of the urinary system and male genital organs—Part A: renal, penile, and testicular tumours. Eur Urol. (2022) 82:458–68. doi: 10.1016/j.eururo.2022.06.016 35853783

[3] Gică N Peltecu G Chirculescu R Gică C Stoicea MC Serbanica AN. Ovarian germ cell tumors: pictorial essay. Diagnostics. (2022) 12:2050. doi: 10.3390/diagnostics12092050 36140449 PMC9498179

[4] Kaatsch P Häfner C Calaminus G Blettner M Tulla M. Pediatric germ cell tumors from 1987 to 2011: incidence rates, time trends, and survival. Pediatrics. (2015) 135:e136–43. doi: 10.1542/peds.2014-1989 25489016

[5] Weil BR Billmire DF. Management of germ cell tumors in pediatric patients. Surg Oncol Clin N Am. (2021) 30:325–38. doi: 10.1016/j.soc.2020.11.011 33706903

[6] Abughanimeh O Teply BA. Current management of refractory germ cell tumors. Curr Oncol Rep. (2021) 23:101. doi: 10.1007/s11912-021-01093-z 34269906

[7] Pfister D Oechsle K Schmidt S Busch J Bokemeyer C Heidenreich A. First-line salvage treatment options for germ cell tumor patients failing stage-adapted primary treatment. World J Urol. (2022) 40:2853–61. doi: 10.1007/s00345-022-03959-8 PMC9712404 35226138

[8] Wang Q Shao X Zhang Y Zhu M Wang FXC Mu J. Role of tumor microenvironment in cancer progression and therapeutic strategy. Cancer Med. (2023) 12:11149–65. doi: 10.1002/cam4.5698 PMC10242329 36807772

[9] Baghban R Roshangar L Jahanban-Esfahlan R Seidi K Ebrahimi-Kalan A Jaymand M. Tumor microenvironment complexity and therapeutic implications at a glance. Cell Communication Signaling. (2020) 18:59. doi: 10.1186/s12964-020-0530-4 32264958 PMC7140346

[10] Paz-Ares LG Ramalingam SS Ciuleanu T-E Lee J-S Urban L Caro RB. First-line nivolumab plus ipilimumab in advanced NSCLC: 4-year outcomes from the randomized, open-label, phase 3 checkMate 227 part 1 trial. J Thoracic Oncol. (2022) 17:289–308. doi: 10.1016/j.jtho.2021.09.010 34648948

[11] Robert C Ribas A Hamid O Daud A Wolchok JD Joshua AM. Durable complete response after discontinuation of pembrolizumab in patients with metastatic melanoma. J Clin Oncol. (2018) 36:1668–74. doi: 10.1200/JCO.2017.75.6270 29283791

[12] Ma W Xue R Zhu Z Farrukh H Song W Li T. Increasing cure rates of solid tumors by immune checkpoint inhibitors. Exp Hematol Oncol. (2023) 12:10. doi: 10.1186/s40164-023-00372-8 36647169 PMC9843946

[13] Santarosa Vieira AG da Silva LS Albino da Silva EC Laus AC Faria TMV van Helvoort Lengert A. Comprehensive microRNA expression analysis of pediatric gonadal germ cell tumors: unveiling novel biomarkers and signatures. Mol Oncol. (2024) 18:1593–607. doi: 10.1002/1878-0261.13617 PMC11161733 38725152

[14] Moreno DA da Silva LS Gomes I Leal LF Berardinelli GN Gonçalves GM. Cancer immune profiling unveils biomarkers, immunological pathways, and cell type score associated with glioblastoma patients’ survival. Ther Adv Med Oncol. (2022) 14. doi: 10.1177/17588359221127678 PMC9791289 36579028

[15] Marques RF Moreno DA da Silva L Leal LF de Paula FE Santana I. Digital expression profile of immune checkpoint genes in medulloblastomas identifies CD24 and CD276 as putative immunotherapy targets. Front Immunol. (2023) 14:1062856. doi: 10.3389/fimmu.2023.1062856 36825029 PMC9941636

[16] Perkins JR Dawes JM McMahon SB Bennett DL Orengo C Kohl M. ReadqPCR and NormqPCR: R packages for the reading, quality checking and normalisation of RT-qPCR quantification cycle (Cq) data. BMC Genomics. (2012) 13:296. doi: 10.1186/1471-2164-13-296 22748112 PMC3443438

[17] Szklarczyk D Kirsch R Koutrouli M Nastou K Mehryary F Hachilif R. The STRING database in 2023: protein–protein association networks and functional enrichment analyses for any sequenced genome of interest. Nucleic Acids Res. (2023) 51:D638–46. doi: 10.1093/nar/gkac1000 PMC9825434 36370105

[18] Korkola JE Houldsworth J Chadalavada RSV Olshen AB Dobrzynski D Reuter VE. Down-Regulation of Stem Cell Genes, Including Those in a 200-kb Gene Cluster at 12p13.31, Is Associated with *In vivo* Differentiation of Human Male Germ Cell Tumors. Cancer Res. (2006) 66:820–7. doi: 10.1158/0008-5472.CAN-05-2445 16424014

[19] Gu Z Eils R Schlesner M. Complex heatmaps reveal patterns and correlations in multidimensional genomic data. Bioinformatics. (2016) 32:2847–9. doi: 10.1093/bioinformatics/btw313 27207943

[20] Alwosaibai K Alruwaii ZI Mashhour M Almsned FM Asraf R Alrsheedy W. Dysgerminomas: germ cell tumors exhibit high expression of PD-L1 and associated with high TILs and good prognosis. Sci Rep. (2024) 14:24191. doi: 10.1038/s41598-024-74192-z 39406772 PMC11480429

[21] Siska PJ Johnpulle RAN Zhou A Bordeaux J Kim JY Dabbas B. Deep exploration of the immune infiltrate and outcome prediction in testicular cancer by quantitative multiplexed immunohistochemistry and gene expression profiling. Oncoimmunology. (2017) 6:e1305535. doi: 10.1080/2162402X.2017.1305535 28507813 PMC5414873

[22] Hinsch A Blessin N Simon R Kluth M Fischer K HubeMagg C. Expression of the immune checkpoint receptor TIGIT in seminoma. Oncol Lett. (2019) 18(2):1497–502. doi: 10.3892/ol.2019.10428 PMC6607271 31423216

[23] Jun E Song AY Choi J-W Lee HH Kim M-Y Ko D-H. Progressive impairment of NK cell cytotoxic degranulation is associated with TGF-β1 deregulation and disease progression in pancreatic cancer. Front Immunol. (2019) 10:1354. doi: 10.3389/fimmu.2019.01354 31281312 PMC6598013

[24] Riggan L Shah S O’Sullivan TE. Arrested development: suppression of NK cell function in the tumor microenvironment. Clin Transl Immunol. (2021) 10. doi: 10.1002/cti2.1238 PMC7797224 33456775

[25] Russick J Joubert P-E Gillard-Bocquet M Torset C Meylan M Petitprez F. Natural killer cells in the human lung tumor microenvironment display immune inhibitory functions. J Immunother Cancer. (2020) 8:e001054. doi: 10.1136/jitc-2020-001054 33067317 PMC7570244

[26] Pouxvielh K Marotel M Drouillard A Villard M Moreews M Bossan A. Tumor-induced natural killer cell dysfunction is a rapid and reversible process uncoupled from the expression of immune checkpoints. Sci Adv. (2024) 10. doi: 10.1126/sciadv.adn0164 PMC11352832 39196934

[27] Dean I Lee CYC Tuong ZK Li Z Tibbitt CA Willis C. Rapid functional impairment of natural killer cells following tumor entry limits anti-tumor immunity. Nat Commun. (2024) 15:683. doi: 10.1038/s41467-024-44789-z 38267402 PMC10808449

[28] Tang X Gao L Jiang X Hou Z Wang Y Hou S. Single-cell profiling reveals altered immune landscape and impaired NK cell function in gastric cancer liver metastasis. Oncogene. (2024) 43:2635–46. doi: 10.1038/s41388-024-03114-0 39060439

[29] Slattery K Woods E Zaiatz-Bittencourt V Marks S Chew S Conroy M. TGFβ drives NK cell metabolic dysfunction in human metastatic breast cancer. J Immunother Cancer. (2021) 9:e002044. doi: 10.1136/jitc-2020-002044 33568351 PMC7878131

[30] Zheng X Qian Y Fu B Jiao D Jiang Y Chen P. Mitochondrial fragmentation limits NK cell-based tumor immunosurveillance. Nat Immunol. (2019) 20:1656–67. doi: 10.1038/s41590-019-0511-1 31636463

[31] Poznanski SM Singh K Ritchie TM Aguiar JA Fan IY Portillo AL. Metabolic flexibility determines human NK cell functional fate in the tumor microenvironment. Cell Metab. (2021) 33:1205–1220.e5. doi: 10.1016/j.cmet.2021.03.023 33852875

[32] Hong P Xu T Xu J Chen W Hu H Chen J. CD24 promotes metastasis and chemoresistance by directly targeting Arf6-ERK pathway in esophageal squamous cell carcinoma. Cancer Lett. (2024) 594:216994. doi: 10.1016/j.canlet.2024.216994 38801885

[33] Altevogt P Sammar M Hüser L Kristiansen G. Novel insights into the function of CD24: A driving force in cancer. Int J Cancer. (2021) 148:546–59. doi: 10.1002/ijc.33249 32790899

[34] Huang S Zhang X Wei Y Xiao Y. Checkpoint CD24 function on tumor and immunotherapy. Front Immunol. (2024) 15:1367959. doi: 10.3389/fimmu.2024.1367959 38487533 PMC10937401

[35] Skowron MA Becker TK Kurz L Jostes S Bremmer F Fronhoffs F. The signal transducer CD24 suppresses the germ cell program and promotes an ectodermal rather than mesodermal cell fate in embryonal carcinomas. Mol Oncol. (2022) 16:982–1008. doi: 10.1002/1878-0261.13066 34293822 PMC8847992

[36] Sloan KE Eustace BK Stewart JK Zehetmeier C Torella C Simeone M. CD155/PVR plays a key role in cell motility during tumor cell invasion and migration. BMC Cancer. (2004) 4:73. doi: 10.1186/1471-2407-4-73 15471548 PMC524493

[37] Lee JB Hong MH Park SY Chae S Hwang D Ha S-J. Overexpression of PVR and PD-L1 and its association with prognosis in surgically resected squamous cell lung carcinoma. Sci Rep. (2021) 11:8551. doi: 10.1038/s41598-021-87624-x 33879814 PMC8058057

[38] Liu W-F Quan B Li M Zhang F Hu K-S Yin X. PVR—A prognostic biomarker correlated with immune cell infiltration in hepatocellular carcinoma. Diagnostics. (2022) 12:2953. doi: 10.3390/diagnostics12122953 36552960 PMC9777148

[39] Marcon F Zuo J Pearce H Nicol S Margielewska-Davies S Farhat M. NK cells in pancreatic cancer demonstrate impaired cytotoxicity and a regulatory IL-10 phenotype. Oncoimmunology. (2020) 9. doi: 10.1080/2162402X.2020.1845424 PMC7714501 33299656

[40] Liu B Arakawa Y Yokogawa R Tokunaga S Terada Y Murata D. PD-1/PD-L1 expression in a series of intracranial germinoma and its association with Foxp3+ and CD8+ infiltrating lymphocytes. PloS One. (2018) 13:e0194594. doi: 10.1371/journal.pone.0194594 29617441 PMC5884516

[41] Woods JK Lidov HG Ligon KL Santagata S Chi SN Yeo KK. PD-L1 and PD-1 expression in pediatric central nervous system germ cell tumors. Modern Pathol. (2022) 35:1770–4. doi: 10.1038/s41379-022-01142-3 36057740

[42] Lobo J Rodrigues Â Guimarães R Cantante M Lopes P Maurício J. Detailed characterization of immune cell infiltrate and expression of immune checkpoint molecules PD-L1/CTLA-4 and MMR proteins in testicular germ cell tumors disclose novel disease biomarkers. Cancers (Basel). (2019) 11:1535. doi: 10.3390/cancers11101535 31614500 PMC6826711

[43] Liston A Humblet-Baron S Duffy D Goris A. Human immune diversity: from evolution to modernity. Nat Immunol. (2021) 22:1479–89. doi: 10.1038/s41590-021-01058-1 34795445

