# Supplementary material for: Immune profiling of pediatric germ cell tumors identifies key cell populations and novel therapeutic targets
Source: Front Immunol. 2025 Jun 20;16:1579948. doi: 10.3389/fimmu.2025.1579948 (PMC12226561; doi:10.3389/fimmu.2025.1579948)
Supplement: Supplementary file 1 [file DataSheet1.pdf]

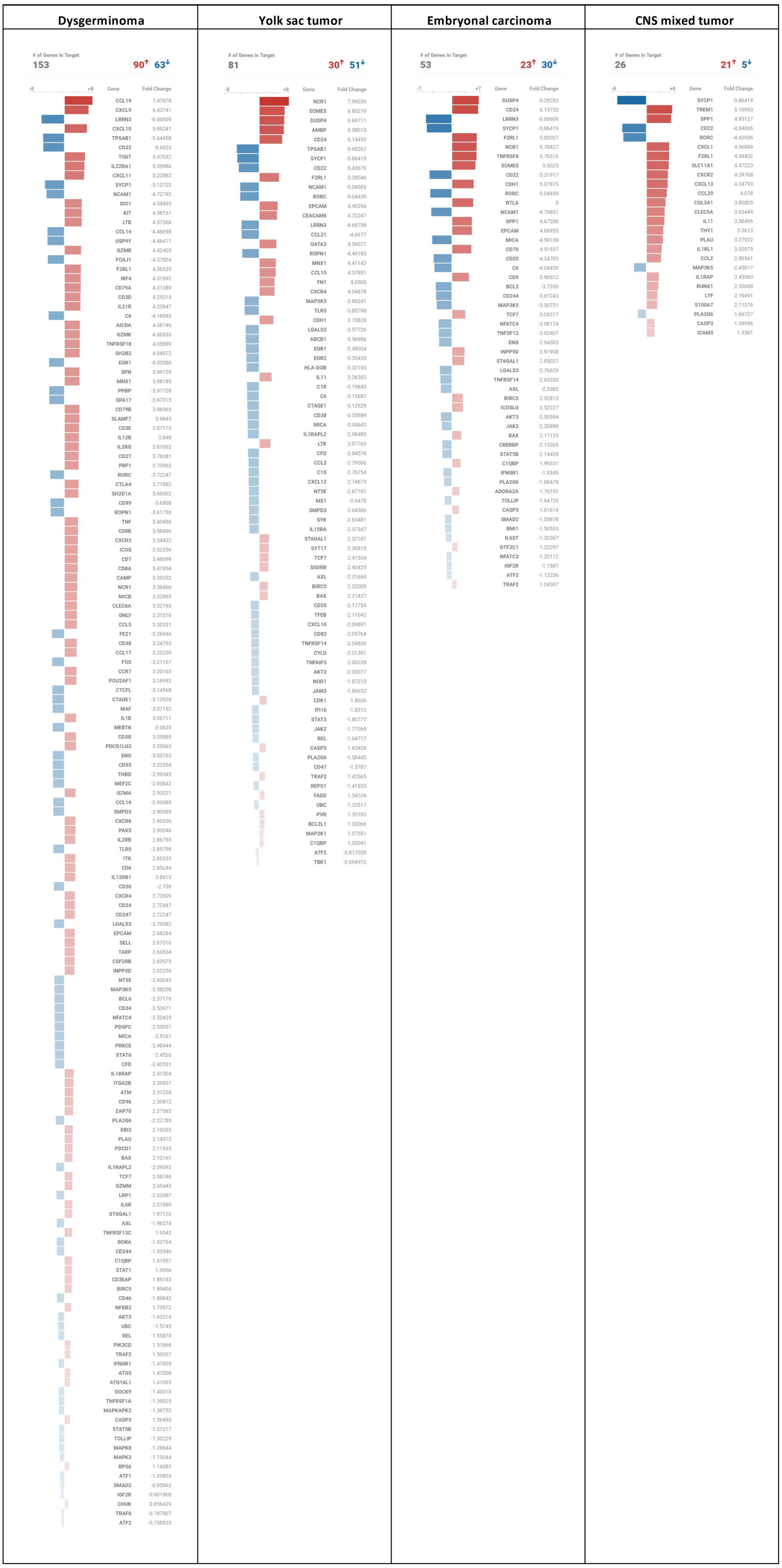

Supplementary Figure 1. Differentially expressed genes (DEGs) in each pediatric germ cell tumor subtype. Bar charts showing the upregulated (red) and downregulated (blue). The total number of DEGs for each subtype is indicated below each panel. Each bar represents an individual gene, with log2 fold change values displayed alongside.
